# Chronic Myeloid Leukemia and Pregnancy: When Dreams Meet Reality. State of the Art, Management and Outcome of 41 Cases, Nilotinib Placental Transfer

**DOI:** 10.3390/jcm11071801

**Published:** 2022-03-24

**Authors:** Elisabetta Abruzzese, Stefano Aureli, Francesco Bondanini, Mariavita Ciccarone, Elisabetta Cortis, Antonello Di Paolo, Cristina Fabiani, Sara Galimberti, Michele Malagola, Alessandra Malato, Bruno Martino, Malgorzata Monika Trawinska, Domenico Russo, Paolo de Fabritiis

**Affiliations:** 1 Hematology, Sant’Eugenio Hospital, ASL Roma 2, Tor Vergata University, 00144 Rome, Italy; trawinskamm@hotmail.com (M.M.T.); paolo.defabritiis@gmail.com (P.d.F.); 2 Obstetrics and Gynecology, Sant’Eugenio Hospital, ASL Roma 2, 00144 Rome, Italy; stefano.aureli@aslroma2.it; 3 Laboratory Medicine, Sant’Eugenio Hospital, ASL Roma 2, 00144 Rome, Italy; francesco.bondanini@aslroma2.it; 4 “Dormant Buds” Association, Obstetrics and Gynecology, San Carlo di Nancy Hospital, 00165 Rome, Italy; mariavita.ciccarone@gemmedormienti.it; 5 Pediatrics, Sant’Eugenio Hospital, ASL Roma 2, 00144 Rome, Italy; elisabetta.cortis@aslroma2.it; 6 Section of Pharmacology, Department of Clinical and Experimental Medicine, University of Pisa, 56126 Pisa, Italy; antonello.dipaolo@unipi.it; 7 Reproduction Pathophisiology and Andrology, Sandro Pertini Hospital, ASL Roma 2, 00157 Rome, Italy; cristina.fabiani@aslroma2.it; 8 Hematology Unit, Department of Clinical and Experimental Medicine, University of Pisa, 56126 Pisa, Italy; sara.galimberti@med.unipi.it; 9 Unit of Blood Diseases and Bone Marrow Transplantation, Cell Therapies and Hematology Research Program, Department of Clinical and Experimental Sciences, ASST Spedali Civili di Brescia, University of Brescia, 25123 Brescia, Italy; michele.malagola@unibs.it (M.M.); domenico.russo@unibs.it (D.R.); 10 Division of Hematology, Ospedali Riuniti Villa Sofia-Cervello, 90146 Palermo, Italy; alessandramalato@hotmail.com; 11 Hematology Unit, Grande Ospedale Metropolitano “Bianchi-Melacrino-Morelli”, 89124 Reggio Calabria, Italy; brunmartin@libero.it

**Keywords:** CML, pregnancy, placental transfer, TKIs, PEG-IFN, conception

## Abstract

The overwhelming success of tyrosine kinase inhibitor (TKI) therapy in chronic myeloid leukemia (CML) patients has opened a discussion among medical practitioners and the lay public on the real possibility of pregnancy and conception in females and males with CML. In the past 10 years this subject has acquired growing interest in the scientific community and specific knowledge has been obtained “from bench to bedside”. Embryological, pharmacological, and pathophysiological studies have merged with worldwide patient databases to provide a roadmap to a successful pregnancy and birth in CML patients. Male conception does not seem to be affected by TKI therapy, since this class of drugs is neither genotoxic nor mutagenic, however, caution should be used specially with newer drugs for which little or no data are available. In contrast, female patients should avoid TKI therapy specifically during the embryonic stage of organogenesis (5–12 weeks) because TKIs can be teratogenic. In the last 15 years, 41 pregnancies have been followed in our center. A total of 11 male conceptions and 30 female pregnancies are described. TKI treatment was generally terminated as soon as the pregnancy was discovered (3–5 weeks), to avoid exposure during embryonic period and to reduce the risk of needing treatment in the first trimester. Eleven pregnancies were treated with interferon, imatinib or nilotinib during gestation. Nilotinib plasma levels in cord blood and maternal blood at delivery were studied in 2 patients and reduced or absent placental crossing of nilotinib was observed. All of the patients were managed by a multidisciplinary team of physicians with obligatory hematological and obgyn consultations. This work provides an update on the state of the art and detailed description of pregnancy management and outcomes in CML patients.

## 1. Introduction

Chronic Myeloid Leukemia (CML), due to its unique oncobiology, is considered a paradigm of targeted therapy efficacy. Since 2000, when tyrosine kinase inhibitors (TKIs) were first used as a therapeutic treatment, prognosis and quality of life (QoL) in CML patients have dramatically improved [1]. After TKIs became the therapy of choice, it quickly was noted that patients in complete cytogenetic response (CCyR) could aspire to the same life expectations as their non-leukemic peers [2]. Encouraged by those results, clinical practice has pursued 2 main objectives: (1) to improve the efficacy of therapy, with the possibility of stopping treatment after achieving stable deep molecular responses (DMR), commonly referred as “treatment free remission” (TFR); (2) to allow the return to as normal a life as possible by managing short- and long-term side effects and improving QoL. Clinicians thus began to face increasing requests concerning the possibility of conception in CML males and carrying a pregnancy to term in CML females, the ultimate expression of “a normal life” [3]. Until a few years ago, this topic was nearly impossible to discuss with patients because of the enormous risks and low probability of success. Recently, however, these discussions have been more frequent as practitioners have started to consider how to manage conception and pregnancy in TFR patients. Analysis of embryological processes and drug toxicity were the first two steps, followed by the creation of databases that collected detailed information about the patient, CML progression, and the child. Understanding these three mutually connected variables is essential to optimally manage conception and pregnancy in CML patients.

In this paper, we will provide an updated review of the topic, together with a description of our experience managing male conception and female pregnancy from 2005 to the present.

## 2. CML Drugs and Conception/Pregnancy

### Interferon α

Before the advent of TKIs, the best available therapy in frontline CML was interferon-alpha (IFN). Its mechanism of action includes immune activation and specific targeting of CML stem cells [4]. IFN reduced the Ph+ clone in approximately 30% of patients and many exhibited extremely stable remissions, which translated to a lower rate of progression and an increased long-term survival [5]. Up to half of those patients remained in remission after discontinuing therapy [6].

IFN treatment does not impact the hypothalamic-pituitary axis [7] in men and animal studies have shown no defects in daily sperm and epididymal sperm concentrations as well as spermatozoa motility [8]. Accumulating evidence in the literature on CML and other illnesses treated with IFNs points to the relative safety of gonadal and pregnancy IFN exposure in terms of maternal and fetal outcomes [9].

Due to production issues, IFNs have been replaced lately with their peghilated form (PEG-IFN) in which the molecule is conjugated with polyethylene glycol (PEG). PEG-IFN has a prolonged half-life and reduced clearance, allowing for single, weekly dose.

## 3. Tyrosine Kinase Inhibitors

TKIs block the constituently active, catalytic activity of the mutant BCR-ABL1 protein, which is responsible for the proliferation, differentiation, and apoptosis of CML cells. However, tyrosine kinases are involved in many signaling pathways and TKIs are not BCR-ABL1-specific. Due to this non-specificity, it was proposed that TKIs might negatively impact reproductive organ function and embryological/fetal development [10].

This hypothesis was analyzed by data from animal studies [11,12,13,14] and patient data obtained from databases [15]. TKIs are neither genotoxic nor mutagenic with the caveat that they can be teratogenic and embryotoxic at high concentrations [16]. Based on these data, there should be no problems in theory in males fathering during therapy. This hypothesis was assessed in ~500 pregnancies described in spouses of males treated with TKIs. In a systematic review Szakacs et al. [17] reported on 428 pregnancies from 374 fathers who conceived without treatment discontinuation under different TKI regimens. Malformations were reported on average of 2.5% of live births, with malformation incidence and type comparable to data from the general population [18]. It is important to note, however, that caution is advised when using ponatinib and other newer TKIs because little or no information on their effects is available.

Normal ovarian function is preserved in animals exposed to TKIs and the increasing number of reported pregnancies in woman treated with TKIs confirm the animal data, although long-term treatment appears to reduce ovarian reserves [19].

Preclinical studies of all licensed TKIs revealed significant embryo and fetal toxicity as reported in TKIs investigator brochures. When used during organ formation (5–12 weeks gestation), TKIs can cause teratogenicity resulting in bone, brain, vascular and/or organ defects [10]. Imatinib is the most studied TKI with more than 300 pregnancies reported. Fetal abnormalities were present in 10–20% of patients treated with imatinib during pregnancy and these data were consistent with abnormalities described in animal studies. Abnormalities observed included craniosynostosis, exomphalos (with an incidence of 3/125 compared to the general population frequency of 1/4000), hemivertebrae, polydactyly, hydrocephalus, myelomeningocele, cerebellar hypoplasia, vascular and organ defects [20]. There are some reports indicating reduced placental crossing of imatinib, suggesting its possible use in selected cases after placental and organ formation have been completed [21].

Pregnancies during nilotinib treatment in women have been described in approximately 60 cases. Abnormalities included one omphalocele and one intrauterine death in twins. Similar to imatinib, nilotinib also does not easily cross the placenta (1/3) and has been used after 16 weeks of pregnancy [22].

Among the 80 pregnancies reported while the mother was on dasatinib, a large percentage (50%) of those with exposure during 1st or more advanced trimesters presented with serious problems including intrauterine death, omphalocele, organ and skeletal malformations. These results are consistent with the observation that dasatinib crosses the placenta and thus can induce problems at any stage of pregnancy [23].

Recently, 16 cases of female patients treated with bosutinib were described. Each of them stopped drug treatment during the 5th or 6th week of gestation and no significant problems were reported [24].

Two pregnancies were reported in the same patient while using ponatinib. In both cases the drug was stopped at the first positive pregnancy test (FPT) at 4 weeks. The first pregnancy ended with a miscarriage at 9 weeks (blight ovum), while the second pregnancy was successful with a spontaneous delivery of a healthy baby boy. The patient has been in “treatment free pregnancy” after stopping treatment [25].

In summary, TKIs should not be used during pregnancy because the probability of serious events exceeds an incidence of 10%. Minimal placental penetration and the data on imatinib and nilotinib use after 16 weeks, in selected cases, suggest the possibility of their cautious administration [26]. Dasatinib should never be used during pregnancy because of teratogenicity and alteration of homeostasis throughout gestation. The US Food and Drug Administration (FDA) therefore assigned TKIs to Pregnancy Category D which states “*there is positive evidence of human fetal risk based on adverse reaction data from investigational or marketing experience or studies in humans, but potential benefits may warrant use of the drug in pregnant women despite potential risks”*.

In contrast to the TKI data, IFN does not appear to be toxic to either the mother or fetus and can be used throughout the pregnancy, including during the first trimester [27]. 

## 4. Fathering in CML

Our center has seen 11 conceptions in 8 female partners of male patients. In one case, sperm was harvested at diagnosis before the start of bosutinib treatment and was later placed directly into the uterus using the medically assisted reproduction (MAR) process of intrauterine insemination (IUI). The pregnancy went to term without problems.

All but 2 patients (one described above and one in TFR) conceived while in therapy. There were 4 imatinib patients, 1 taking nilotinib and 1 being administered ponatinib. Moreover, 2 of the 4 imatinib patients generated multiple pregnancies (2 and 3). The partner of one imatinib patient chose to undergo a voluntary abortion for non-CML related reasons. The 10 pregnancies carried to term did not present any particular problems or malformations. The girl born as a result of the IUI initially presented with mild language retardation, however she, now 10 years old, has recently improved to near normal function with good school performance in the past 2 years. Details and outcome are outlined in Table 1.

## 5. Pregnancy with CML: Our Center’s Experience

Since 1990, our unit has had a multidisciplinary group that manages fertility and pregnancy in hematology patients. The group is coordinated by hematologists and has frequent interactions with obgyn physicians. One specific obgyn oversees each of our family planning patients including those who come to the high-risk pregnancy clinic already pregnant, and those in the gynecological hormone clinic who are planning a pregnancy after or during a hematological treatment or in early menopause. When needed, andrologists, endocrinologists and neonatologists/pediatricians are consulted. Patients also have access to our recently approved infertility clinic that provides evaluation, ovum harvest and MAR including IUI and in vitro fertilization (IVF), all free of charge within the Italian national health system. Most pregnancies reviewed by the group before 2010 involved lymphomas, acute leukemias, myeloproliferative disorders and non-neoplastic hematological disorders such as hemoglobinopathies and thalassemia [28].

Since 2007, we have followed 22 pregnant patients affected by CML. Five were diagnosed with CML at onset of pregnancy. Two decided to stop the pregnancy (voluntary termination of pregnancy, VTP), while two carried the pregnancies to term, and one is ongoing. A total of 17 patients underwent 25 pregnancies (1 set of twin). Of those, 20 babies were born, 1 pregnancy is ongoing and there were 4 abortions (1 VTP and 3 miscarriages before week 10).

All pregnancies were spontaneous except the one ongoing. That patient underwent MAR using intracytoplasmic sperm injection (ICSI), stopping nilotinib at the FPT. The patient is 26-year-old G1P0, diagnosed 2 years earlier in MR 4. She lost MR3 at the beginning of second trimester and started peg-IFN at 20 weeks. She is now in MR3 and in her 26th week of gestation with pregnancy proceeding normally. Although it would be better to harvest oocytes before starting any treatment, our patient’s oocytes were collected after 2 years of nilotinib treatment. The 3 reported cases of oocyte retrieval in CML treated patients had a washout period ranging from 19 days to 6 months [19]; however, considering the physiology of ovulation and the literature on natural conceptions during TKI therapy, we thought this was not necessary.

Nearly all patients in treatment stopped TKI at the FPT, except in 3 cases in which imatinib was stopped 6 weeks before pregnancy, and in 1 other patient in which nilotinib was switched to IFNα before her 2 pregnancies started [29]. One patient was in TFR.

## 6. Management of Pregnancy at CML Onset

Five patients were diagnosed with CML at the time of pregnancy onset. All patients were counseled concerning risks and possibilities. Furthermore, 2 patients (35 years old, G4P3, and 26 years old G1P0) decided to voluntarily terminate the pregnancy, while the other 3 patients (27, 31 and 37 years old), 2 in their first pregnancy (G1P0), 1 who had a previous spontaneous abortion, pursue the gestation.

The 27-year-old patient, diagnosed at 6 weeks of pregnancy with a WBC of 80,000, started IFNα in the 9th week of gestation after reaching the threshold of 100,000 WBC. The patient achieved complete hematologic remission with transcript levels remaining above 50% IS. A healthy baby girl was born at 38 weeks by caesarian section (CS) and her mother switched to dasatinib after the baby received colostrum for one week.

The 31-year-old woman, presenting with a p190 transcript, had a stable WBC (28 to 42,000) throughout the pregnancy and, thus, it was not necessary to give any CML drug. A healthy baby boy was born after 39 weeks by CS. This patient also started on dasatinb a few weeks after delivery. At the time of this writing, both babies are 3 years old and growing normally, and the mothers remain on their first line therapy in deep molecular response.

The last patient, the 37-year-old, was recently diagnosed as having CML at 10-week pregnancy with 22,000 WBC but 861.000 platelets, rapidly increasing to 1,200,000. She was also thrombophilic with heterozygous mutation of Factor V Leiden and had lost a baby in a previous pregnancy during first trimester. For this reason, even if placed under low molecular weight heparin and cardioaspirin, we decided to start PEG-IFN at 17 weeks. She is now at 26 weeks of gestation, is being treated with interferon and has achieved complete hematologic remission.

This experience, however limited, confirms the ability, under certain conditions, of successfully handling a fragile situation without compromising the present and the future outcome of either the mother or child.

## 7. Management of CML Therapy during Pregnancy

Twenty-five pregnancies were reported and followed by our team in patients already diagnosed and treated for CML. One is ongoing and is not included in this discussion, as it has been already mentioned above. 

There were 3 spontaneous abortions and 1 VTP, the latter in a 40-year-old G1P0 who discovered pregnancy at 8 weeks gestation during imatinib therapy. Patients who suffered spontaneous abortions subsequently succeeded in getting pregnant. The remaining 20 pregnancies went to term. Details are presented in Table 2.

Most pregnant patients stopped TKI at FPT (3–5 weeks). A total of 3 patients stopped TKIs prior to becoming pregnant: 1 patient 6 weeks earlier, while another patient who had two pregnancies stopped TKI treatment and switched to IFNα 5 months and 1 month, respectively, prior to conception to conceive free of TKIs; 1 patient was in TFR since her previous pregnancy (patient #7 Table 2) and never resumed TKI.

Nine patients were treated at some time during pregnancy, two with nilotinib, one with imatinib, and the others with IFN.

One 25-year-old patient (G1P0), having been treated with ponatinib and IFN, stopped ponatinib at 4 weeks (FPT). This patient continued with αIFN, however, she had a spontaneous abortion at 9 weeks. 

One 36-year-old patient (G1P0), treated in second line with nilotinib and in MR3, discovered her pregnancy at 7 weeks and her TKI treatment was replaced by IFNα. However, she rapidly lost her molecular and cytogenetic responses and was re-placed at 21 weeks on nilotinib at a lower dosage (400 mg daily). She spontaneously delivered a healthy baby boy-now 4 years old-at 39 weeks in MR4.

The second patient treated with nilotinib (36 years old, G2P0) stopped therapy at FPT at 4 weeks while in MR4. The MR4 response was lost between the 1st and the 2nd trimester with transcript levels above 2% IS and therapy with nilotinib was restarted at 23 weeks at full dose (600 mg daily). She spontaneously delivered a healthy baby boy at 41 weeks in MR4.

The 3rd patient, 34 years old G1P0, was treated with TKI during pregnancy while in MR3. She stopped imatinib at 3 weeks but rapidly lost molecular, cytogenetic, and hematologic remission during the 1st trimester. Imatinib was restarted at 20 weeks, and she spontaneously delivered a healthy baby boy at 34 weeks due to an early rupture of membranes while in MR2 (0.8% IS). The mother switched to dasatinib shortly after delivery and is now in MR4. At the time of this writing, her child is a healthy 1 year old male.

The management skills acquired in our Center over the years of overseeing hematological diseases during pregnancy has enormously helped in handling CML and pregnancies/conception. A clinic dedicated to CML patients, with expert clinicians and facilities able to rapidly obtain PCR results are key elements to optimally manage CML and gestation.

Each case of pregnancy must be individually considered. Kinetics of transcript changes, tumor burden and CML status (onset, remission, line of therapy, etc.), and the stage and the course of pregnancy must guide the decision to start or re-start therapy. Our experience has evolved throughout the years, from initially being more conservative (e.g., no treatment at all until hematologic response was lost) to now being more comfortable prescribing an early intervention (e.g., initiating IFN to maintain response after stopping TKI or as soon as the transcript starts to rise, or TKI re-start as soon as MR3 is lost).

## 8. Nilotinib Placental Transfer

**Figure 1:**
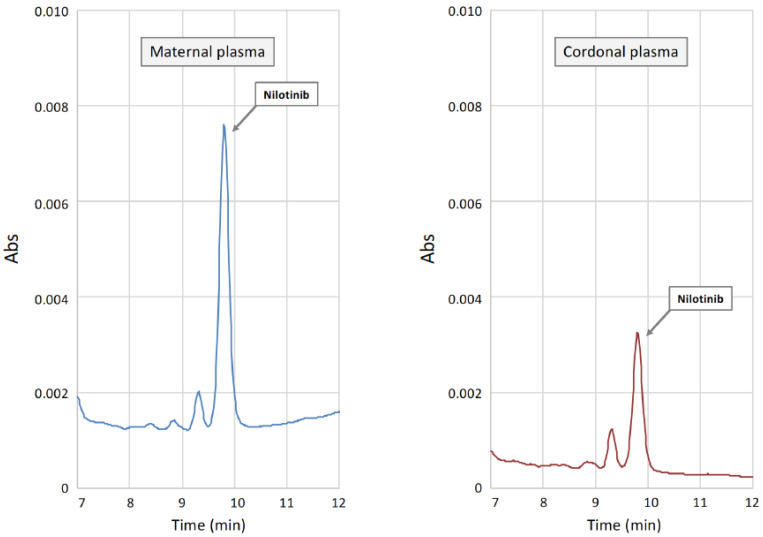
Representative chromatograms obtained from the UV-HPLC analysis of plasma samples obtained from maternal (**left**) and cord (**right**) blood in patient # 10 showing little (~0.33%) transfer of the drug.

The 2 patients treated with nilotinib at term were studied for placental transfer at delivery. Nilotinib concentrations in plasma samples were measured by a validated chromatographic method with UV detection. Maternal and cord blood samples were centrifuged, and the resulting plasma was processed using a commercial kit together with calibration standards and quality controls (Chromsystems, Munich, Germany). The chromatographic separation of nilotinib and the quantitation of drug peaks areas in samples was carried out with the same kit. The final concentrations of nilotinib in patients’ samples was obtained by a linear calibration curve obtained through the analysis of standard samples (Chromsystems, Munich, Germany). Results of the chromatographic analysis of maternal plasma demonstrated the presence of nilotinib at concentrations of 1.2 and 1.5 mg/L, respectively, values higher than the minimum threshold concentration for efficacy (0.465 mg/L), and equal to the mean value of nilotinib plasma concentration measured in patients taking the standard daily dose of the drug [30,31,32]. Interestingly, only one of the two cord plasma samples had a measurable plasma concentration of nilotinib. In the patient with the higher maternal plasma concentration (1.5 mg/L) and taking the full dose (600 mg) of the drug, nilotinib was not found in the cord blood. The other patient, that was assuming nilotinib at the reduced dosage (400 mg), revealed a plasma concentration of 1.2 mg/L. Cord blood nilotinib search was positive, and a value of 0.380 mg/L, approximately 1/3 of the maternal sample (Figure 1, left), similar to what has been reported in the literature [22].

Several factors could be responsible for the discrepancies between the present nilotinib values in cord blood samples. Among TKIs, the pharmacokinetics of nilotinib appears not to be affected by the variable expression/activity of transmembrane transporters [33,34] even if those transporters are widely expressed at the placental barrier [35]. Moreover, a different time interval between drug intake and cord blood withdrawal at delivery could explain the difference in plasma drug concentrations. Finally, the contamination of the cord blood with maternal blood could be a possible explanation for the presence of nilotinib at quantifiable concentrations in one of the two cord samples.

## 9. Discussion

In the past 10 years, together with the improved prognosis and QoL, we have seen an increasing number of literature reports on pregnancy/conception in CML patients. We approached the subject from two points of view: CML biology and patient data.

Regarding the biology, the first step has been to understand the effects of treatments on the embryology and homeostasis during all pregnancy stages. This includes a thorough analysis of all TKI preclinical animal studies with regard to gonadal genotoxicity and mutagenicity as well as conception toxicity and potential teratogenesis.

TKIs are neither genotoxic nor mutagenic, however, they can be teratogenic at standard doses causing abnormal development of the embryo or fetus. The 2 most susceptible periods when exposure to a teratogenic agent has the greatest likelihood of producing a malformation are: (1) the embryonic period from pregnancy week 5–6 to week 10 during which organogenesis takes place; (2) the early fetal period, specifically weeks 11–12 [36,37]. Beyond week 16, when the placenta is formed and organs are developed, the use of TKIs such as imatinib and nilotinib that have a reduced placental crossing can limit potential risks. In contrast, dasatinib can have detrimental effects on fetal growth, development and homeostasis from this stage onwards including during later pregnancy stages (2nd–3rd trimester) because it passes across the placenta [38,39]. It is important to note that drugs alone may not produce congenital abnormalities. The principle of teratogenesis recognized by Wilson in 1959 states that genetic and epigenetic factors of the embryo and the mother, exposure timing and duration, and dosage all influence the ultimate outcome. Observational studies, through case reports or case series, are the usual methods to determine the impact of a drug exposure in humans.

Of the few case reports published around 2005, 1 of the first was by Ali et al. [40] reporting on a woman who, after 5 months of imatinib treatment and already in CCyR, discovered she was 8 weeks pregnant. Imatinib was stopped immediately and her pregnancy and CML situation were closely monitored. The patient remained off treatment for the duration of the pregnancy and as expected, had a cytogenetic relapse of CML at 7 months gestation, 5 months after discontinuation of imatinib therapy. She presented with a hematological relapse at 8 months gestation. Neither the cytogenic nor hematological relapses impacted the pregnancy, and she delivered a healthy girl.

Pye et al. in 2008 [41] was the first to describe malformations in offspring from 180 CML patients, 145 for whom imatinib exposure information and outcome details were available. Among those pregnancies, 103 were exposed in the first trimester, 4 during first and second trimesters, and 38 continue drug treatment throughout the pregnancy. Congenital abnormalities were reported in 9 infants, and 3 elective terminations. Moreover, 10 of the 12 infants with abnormalities were exposed to imatinib during the first trimester which includes organogenesis (information was unavailable for the remaining 2 infants). The incidence and the type of abnormalities greatly exceeded the predicted abnormality rate in the general population. Furthermore, the infants had a combination of very similar, quite complex defects which would have been unlikely to occur by chance, suggesting a direct effect of the drug.

Recent reports include data from several individual medical centers. Assi et al. from MD Anderson described the outcome of 51 pregnancies in 37 patients, 30 females and 7 males, with a focus on the management of patients throughout the pregnancy [42]. The 7 male patients did not stop TKI treatment and outcomes were normal. All female patients discontinued TKI at pregnancy confirmation or earlier. The researchers also examined planned and unplanned pregnancies and found that TKI interruption resulted in a transient loss of molecular response that could be regained rapidly post-partum after treatment resumption. An Indian study using data from 58 males and 46 female cases collected since 1998 included pre-imatinib and post-imatinib data [43]. Male patient offspring were normal, while in the 10 female patients who continued imatinib until delivery, there was one baby born with omphalocele and one with craniosynostosis. The 2 infants underwent successful surgeries and are now 6 and 2.5 years old with normal development.

To obtain detailed case series information concerning pregnancy, CML and newborns, multicenter observational prospective and retrospective studies have recently been carried out. The two largest studies include the GIMEMA database in which Italian centers have collected data on both conceptions in male patients and pregnancies in female patients, and the ELN registry that has gathered data worldwide on female pregnancies.

A total of 150 patients (85 male; 65 female) have been examined in the GIMEMA study and preliminary results from this study were presented at ASH 2018 for 171 pregnancies. Median age at conception for both groups was 33 years old. Furthermore, 8 of the 171 pregnancies were the result of MAR procedures (5 males, 3 females); 11 abortions in 2 male conceptions (both elective) and 9 female pregnancies (5 elective) were not included in the 171 pregnancies. All abortions occurred during the first quarter of pregnancy and were not related with known congenital malformations or genetic defects. Most female patients stopped TKI treatment at FPT, although 10 continued to receive treatment during the 2nd–3rd trimesters (2 imatinib, 2 nilotinib, 6 IFN). Delivery occurred at a median of 40 weeks in the partners of male patients and 39 in female patients, and vaginal delivery in female patients was equivalent to the Italian national rate of 2/3 (68%). As expected only 29% of female patients could breast feed. All patients who lost their MR response and restarted therapy regained their remission. The following abnormal issues were reported: an otherwise normal baby with a macrocephalic head; 1 baby with language problems resolved with tutoring; 1 child with autism; 1 rheumatoid arthritis case; 1 report of celiac disease [15]. Full manuscript of GIMEMA registry is in preparation, and cases presented here will be included.

The ELN international registry collected data on more than 250 female patients from 17 European and non-EU countries. Preliminary data presented at EHA and ASH in 2019 highlighted the fact that CML patients are able to pursue a normal life, including planning a family. Most patients (75%) conceived while on a TKI with early interruption at FPT prior to organ formation; outcomes in those patients were not associated with congenital abnormalities.

These studies indicate that CML patients diagnosed during pregnancy can delay treatment without jeopardizing future CML control. Leukemic burden is an important factor in deciding to treat a pregnant patient, and the timing of the rise in transcript levels should guide treatment decisions. Concerning drugs, data from the ELN study on IFN (45 patients) and TKIs (66 patients) confirmed that IFN or PEG-IFN can be used throughout the pregnancy and can help to induce and maintain hematological remission or preserve molecular remission after TKI cessation.

IFN can be successfully prescribed in its peghilated form. PEG-IFN has been used during pregnancy in myeloproliferative disorders (e.g., essential thrombocytemia) with similar outcomes compared to standard IFN formulation [44]. Only one case report has been found in the literature for CML and few cases included in the ELN registry [45]. The 2 cases presented here, both at 26 weeks with pregnancy ongoing, add to these data.

Recent recommendations on CML and pregnancy management have been described after direct experience and analysis of published data [46]. The data reported in this paper confirm that in males TKI treatment does not seem to impact on fertility and conception. In contrast, female patients need to stop TKI therapy as soon as possible during the first 5 weeks of gestation. It is important to note that both planned and unplanned pregnancies as well as CML onset during pregnancy can be managed.

A strict follow up of CML and course of gestation as well as post-natal development of the baby is mandatory to optimally manage all situations. Considering this, a multidisciplinary medical team handling reproduction and pregnancy in CML patients is a winning approach.

## Figures and Tables

**Table 1:** Conception in male CML patients.

PT Number	Age @ Conception	Number of Conceptions	TKI Type	Δt TKI	Delivery (S/CT)	Week of Delivery	Age Kid (years)
1	39	1	NILO	2 months	S	40	
2	27	2	IMA	10 years	S	39	10
	28		IMA	11 years	S	38	9
3 (MAR)	42	1	none		CT	36	10
4	37	1	IMA	6 months	/	/	/
5	42	3	IMA	5 years	S	39	11
	43		IMA	6 years	S	41	10
	51		IMA	14 years	S	40	2
6	40	1	PONA	3 years	CT	40	4
7	34	1	IMA	11 years	S	40	1
8	33	1	TFR post IMA	8 years	S	39	13

PT number: patient number; Age @ conception: father age at conception; Number of conceptions: total number of conceptions after chronic myeloid leukemia (CML) diagnosis; TKI type: tyrosine kinase inhibitor (TKI) therapy at conception; Δt TKI: amount of time the latter TKI was used; Delivery (S/CT): type of delivery (S = spontaneous, CT = cesarean section); Week of delivery: delivery time (in weeks); Age kid (years): age of the child; in years; MAR: Medically Assisted Reproduction.

**Table 2:** Pregnancies in female CML patients.

PT Number	Age @ Pre	Date CML diag	Number of Children	TKI @ Pregnancy	TKI Stop @ Pregnancy	MR t0	MR t3	MR t6	MR t9	TX during Pregnancy/TX Type	Delivery (S-CT)	Week of Delivery	Breast Feeding	Age Kid (Dec21)
1	36	July 2001	1	IMA	4 weeks	0.0063	1.7	1.9	0.93	**IFN 14 weeks**	CT	36	NO	3
2	36	December 2011	0 ABORTION	NILO	3 weeks	0.018				no				
	36	December 2011	1	NILO	4 weeks	0.0033	1	1	0.032	**NILO 23 weeks**	S	41	NO	3
3	34	November 2010	1	DASA	4 weeks	0.0859	0.088	0.086	0.081	**IFN 25 weeks**	S	40	NO	5
4	28	June 2019	1	NILO	7–8 weeks	1.11	0	13	16	**IFN 12 weeks**	CT	37	UNK	6
5	33	November 2003	1	IMA	1 weeks	0	0.00044	5.8	7.3	no	CT	35	UNK	10
6	40	April 2004	0 VTP	IMA	5 weeks	0.03				no				
7	32	April 2006	1	DASA	3 weeks	0	0.0049	0.0079	0.0006	no	S	40	NO	4
	34		1	NO	TFR	0.0053	0.0077	0.0013	0.0089	no	S	39	NO	2
8	34	October 2007	1	IMA	4 weeks	0.0088	0.027	0.0032	0.03	no	S	40+1	YES	8
	36		1	NILO	4 weeks	0.0013	0	0.001	0.0054	no	S	39	YES	5
9	30	January 2010	1	DASA	4 weeks	0.019	0.012	0.0053	0.0066	no	S	36	NO	6
	32		1	DASA	4 weeks	0.0059	0.0032	0.015	0.045	no	S	36	YES	4
10	36	November 2003	1	NILO	7 weeks	0.012	3.8	1.5	0.001	**IFN 7 weeks**	S	39	NO	4
							15.20 a 21 weeks			**NILO 21 weeks**				
11	35	February 2006	1	IMA		0.001	6.4	0.18	0.22	**IFN 13 weeks**	S	39	NO	4
12	40	March 2013	0 ABORTION	NILO	1 weeks	0.023				no				
	42		2	NILO	4 weeks	0	0.05	0.12	0.0048	no	CT	40	YES	4
13	31	March 2006	1	NILO	20 weeks before	0	0	0	0	no	S	42	YES	8
	36		1	NILO	4 weeks before	0	0	0	0	no	S	36	NO	3
14	35	November 2014	0 ABORTION	PONA+INF	4 weeks	0.0046				**IFN**				
	36		1	PONA	4 weeks	0.0062	0.0054	0.0095	0.008	no	S	40	YES	1
15	34	February 2003	1	IMA	3 weeks	0.03	56	2	0.8	**IMA 20 weeks**	S	34	NO	1
16	40	September 2003	1	DASA	6 weeks before	0	0	0	0	no	S	35	YES	7
17	26	May 2018	1	NILO	4 weeks	0.0055	0.12	0.084	NA	**P-IFN 20 weeks**				ongoing
18	35	February 2008	0 IVG	NO		NA				no				
19	31	December 2017	1	NO		58				no	CT	39	NO	3
20	26	November 2007	0	NO		NA				no				
21	27	August 2017	1	NO		87	72	88	85	**IFN 9 weeks**	CT	38+2	NO	3
22	37	September 2021	1	NO		75	ND			**P-IFN 17 weeks**				ongoing

PT number: patient number; Age @ Pre: mother age at conception; Date CML diag: date of chronic myeloid leukemia (CML) diagnosis; Number of children: total number of pregnancies after CML diagnosis; TKI @ pregnancy: last used tyrosine kinase inhibitor (TKI) type; TKI stop @ pregnancy: gestational week of tyrosine kinase inhibitor (TKI) stop; MR t0: molecular response at early pregnancy; MR t3: molecular response at first trimester; MR t6: molecular response at second trimester; MR t9: molecular response at delivery; IS%: Molecular response results expressed in IS%; TX during pregnancy/TX type: therapy during pregnancy/type of therapy; gestational week at which therapy was started; Delivery (S-CT): type of delivery (S = spontaneous, CT = cesarean section); Week of Delivery: delivery time; Breast Feeding: breast feeding of the baby; Age Kid (Dec21): age of the child (updated on 31 December 2021); MAR: Medically Assisted Reproduction; Yellow cases refer to patients diagnosed with CML during pregnancy. Patients treated during pregnancy are evidenced in bold and red.

## Data Availability

Data available on request due to restrictions (privacy).
